# Barriers to Reducing Children’s Screen Time and Associated Factors Among Mothers of Under-Five Children in Woliso City Administration, Oromia Ethiopia: Community-Based Cross-Sectional Study

**DOI:** 10.2196/100021

**Published:** 2026-10-08

**Authors:** Ababo Demeke, Wubishet Gezimu

**Affiliations:** 1 Department of Nursing, College of Medicine and Health Science, Dilla University, Dilla, 419, Ethiopia, 251 0920305115; 2 Department of Nursing, College of Health Science, Mattu University, Mattu, Ethiopia

**Keywords:** parental barriers, screen time, predictors, children, mothers, associated factors, under-five children, Woliso City, Ethiopia

## Abstract

**Background:**

Screen time (ST) is the time spent using screen-based devices and has become a global health concern. Excessive ST negatively impacts children’s health and development; however, it has been increasing dramatically among young children. Parents are the primary caregivers responsible for monitoring children’s ST. Therefore, identifying potential barriers and their attributes is fundamental to taking early action.

**Objective:**

The study aimed to assess barriers to reducing excessive ST and the factors associated with these barriers among mothers of under-five children in Woliso City Administration, Central Ethiopia.

**Methods:**

A community-based cross-sectional study was conducted from January 10, 2024, through February 29, 2024. A systematic random sampling technique was used to select 423 mothers of under-five children. EpiData (version 3.1) was used for data entry, and SPSS (version 20) was used for analysis. Linear regression analysis was used to identify factors associated with barriers to reducing ST. The multiple linear regression assumptions were checked, and statistical significance was declared at *P*<.05 with 95% CIs.

**Results:**

A total of 423 participants were involved in this study (a 100% response rate). The mean score of barriers among mothers was 4.28 (SD 1.04). Educational status of the mother (β=−0.130, 95% CI −0.255 to −0.004), child’s ST (β=0.369, 95% CI 0.328-0.409), parental attitude (β=−0.382, 95% CI 0.264-0.499), parental perceptions of the effect of ST on cognitive well-being (β=0.021, 95% CI 0.009-0.034), parental restrictive practices regarding ST (β=−0.011, 95% CI −0.004 to −0.018), and availability of public facilities (β=0.849, 95% CI 0.759-0.940) were shown to have a significant association with parental barriers.

**Conclusions:**

Most mothers of under-five children experienced 4 barriers to reducing ST. The study also identified 6 factors associated with barriers to reducing ST, including level of education, attitudes toward ST, restrictive practices, perceptions of cognitive effects, children’s ST, and lack of public facilities. Enhancing access to public spaces, implementing educational programs, and developing community initiatives are recommended to overcome these barriers and support healthy screen habits.

## Introduction

Screen time (ST) refers to a person’s time spent using screens or digital devices such as smartphones, tablets, televisions, video games, computers, or wearable technology [1-3]. According to the World Health Organization (WHO), children under the age of 2 years should not have any ST, whereas those aged 2 to 5 years are recommended to limit their ST to a maximum of 1 hour per day. Individuals exceeding these guidelines are classified as having excessive ST [2].

Recently, ST has emerged as a global health concern [4]. The duration of exposure to screen-based devices has been progressively increasing over time [1]. Notably, a substantial proportion of young children are currently exceeding the WHO’s recommendations for ST [5]. For instance, in Singapore, children aged 2 years have an average ST of 2.4 (SD 2.2) hours per day [6]. In Canada, the mean ST for children is 1.4 hours per day at age 5 and 1.5 hours per day at age 3 [7], while in Ontario, 13.6% and 43.5% of children exceed 2 hours per day and 1 hour per day, respectively [8]. Furthermore, in the United Kingdom, 75% of children aged 12 months exceed zero ST [9]. In addition, excessive ST is observed in Latin America (47%), Brazil (66.05%), and Malaysia (91.4%), where children spend significant amounts of time on screens [10-12]. Specifically, in Australia, children at 6 months of age are exposed to screens for an average of 1 hour and 16 minutes per day, which increases to 2 hours and 28 minutes by 24 months, with some children exceeding 3 hours per day [13]. Moreover, in Japan, 29.4% of children aged 18 months and 24.5% of children aged 30 months watch television for 4 or more hours per day [14]. In Portugal, about 85% of children under 2 years and 80% of infants aged 6 to 12 months are exposed to screens daily, with 79% spending up to 1 hour per day [15]. Similarly, a recent study in Ethiopia reported that 75.9% of under-five children had excessive ST, underscoring that this global concern is also highly prevalent in local Ethiopian settings [16].

Excessive ST causes various problems in children. It compromises both physical and cognitive development in young children and is positively associated with obesity, sleep problems, depression, and anxiety [17]. It generally leads to delays in all developmental domains, including physical, cognitive, mental, social, and scholastic [18,19]. Physically, it contributes to obesity [3,20,21], a sedentary lifestyle [3], disturbed sleep [22-24], headaches [3], eye strain [3,25], and musculoskeletal pain, such as in the neck, back, and wrists [3]. Mentally, it is linked to communication delays, including expressive speech delay [3,19,26,27], repression or delay in cognitive and motor development [19,27,28], hyperactivity-inattention [3,14], autism spectrum disorder [29], aggressive behavior [3], antisocial behavior [14], a desire for instant gratification [3], and poor concentration [3,7]. Additionally, it leads to fear of missing out, fear of being left out, media addiction, self-harm, anxiety, and depression [3]. Socially, excessive ST causes delays in socioemotional development [19], reduced socialization, and increased social anxiety [3]. Scholastically, it is associated with decreased academic performance [3]. These adverse effects underscore the necessity for parents to limit their children’s ST to recommended levels [1-3,23].

Parents play a crucial role in shaping their child’s screen usage, as they are their child’s first teachers [4,30,31]. They have significant control over their child’s health behaviors during the younger years [32], and children tend to adopt habits established in early childhood throughout their lives [22]. Parents should act as role models for healthy screen use, limit their own media use, and model online etiquette. Attentive parenting requires face time away from screens [3,23]. While both parents influence children’s screen use, this study focused exclusively on mothers because they are the primary caregivers in most Ethiopian households, particularly during the early years of child development. Mothers typically spend more time with under-five children than fathers. Therefore, examining maternal perspectives provides the most relevant insights into parental barriers to reducing ST in this context.

Parents face multifaceted challenges in monitoring their children’s ST. Common barriers include societal pressure to use screens, children’s enjoyment of ST, busy family schedules, poor weather limiting outdoor play, and the perceived need for ST for educational purposes [33]. Additionally, parents often face a lack of time, parental fatigue [34], and safety issues [35]. A Reddit content analysis during the COVID-19 pandemic highlighted various barriers, including competing work and in-home obligations, using screens to occupy children during travel, children’s screen use with other caregivers, offering ST while parents needed rest, pandemic-related changes in routine, and using screens to encourage necessary behaviors [36]. A study from Selangor, Malaysia, reported an overall mean score of 3.51 (SD 0.83) for parental barriers [37].

Despite rapid urbanization and increasing access to digital devices, there has been limited information about the concept of ST and parental barriers in supervising their children’s screen use. Yet there are no national guidelines or structured interventions to help parents manage screen exposure in early childhood. Moreover, caregiving practices, including reliance on screen devices to calm a baby or to complete household chores, may complicate efforts to reduce screen use. Socioeconomic disparities and limited access to public facilities further exacerbate barriers to reducing ST.

Identifying and understanding barriers to reducing ST is crucial for developing effective interventions to promote healthy screen usage among children under-five. However, there has been limited information available about parental barriers to reducing children’s ST and associated factors. As this was the first study of its type in Ethiopia, it may shed light on the parental obstacles to monitoring their children’s ST, and the information obtained from this study may be used as a foundation for future studies in the field.

Accordingly, this study aimed to answer the following research questions:

1. What is the mean level of barriers to reducing children’s ST among mothers of under-five children in Woliso City Administration?
2. What factors are associated with barriers to reducing children’s ST?

## Methods

### Study Design and Setting

A community-based cross-sectional study was conducted from January 10, 2024, through February 29, 2024, in Woliso City Administration. The city is in Oromia Regional State, about 114 km southwest of Addis Ababa, the capital city of Ethiopia. Ethiopia is rapidly shifting from an agrarian lifestyle to urbanization [38]. The country is also undergoing a digital revolution as a result of the national initiative “Digital Ethiopia,” which is playing a crucial role in access to digital devices and increased usage [39]. As a result, about 85.4 million and 28.6 million people are actively using mobile phones and the internet, respectively [40]. In particular, about 94.8% of parents in Woliso City had screen devices, of which 99.4% were mobile phones [16].

Regarding ST and the physical environment, about 54.5% of Ethiopian children and adolescents adhere to the sedentary recreational ST guidelines, whereas only 43% of children and adolescents have access to physical activity settings [41,42]. In Woliso City, only 26.7% of parents reported having available playgrounds, and about 56.8% of children had outdoor playtime of more than 1 hour [16].

Generally, the study setting (Woliso City) has 4 administrative kebeles (lower administrative units)—namely Ayetu, Egersa, Burka Gudina, and Hora—with an estimated 15,476 households. As of 2014, the estimated total population of the city was 118,000, including 19,389 children under the age of 5 years.

### Study Participants and Eligibility Criteria

All mothers of under-five children living in the Woliso City Administration were the source population. All mothers of under-five children who were permanent residents of the city and willing to participate were included in the study. However, mothers who did not have a screen device or whose children were blind were excluded from the study.

### Sample Size Determination and Sampling Method

The sample size needed for this study was determined using the single-population proportion formula, considering a 95% CI level, a 5% margin of error, and a 50% proportion of occurrence. After adding a 10% nonresponse rate, a sample size of 423 was obtained and allocated proportionally across kebeles. A systematic random sampling technique was used, targeting every 36th household. The first household was selected by lottery method. In each selected household, mothers or guardians of under-five children were approached and invited to participate. Data collectors explained the study objectives, obtained informed consent, and administered the questionnaire to eligible respondents.

### Data Collection Instruments and Techniques

Four data collectors and 1 supervisor conducted the data collection using a validated, structured, and pretested interviewer-administered questionnaire adapted from a previous study [37], after translating it into the local languages (Afan Oromo and Amharic). The questionnaire contained sociodemographic information, child-related factors, parental factors, parental barriers to reducing ST, home environment–related questions, and neighborhood environment–related questions.

Sociodemographic factors include information related to family size, age of the mother, age of the father, marital status, educational status of both parents, occupation of both parents, religion, ethnicity, number of rooms in the house, and monthly income.

Regarding child-related factors, the questionnaire included age, sex, number of children, ST in minutes, and childcare facility. A child’s average daily ST, reported by parents, was calculated by multiplying weekday ST by 5 and weekend ST by 2, then dividing the sum by 7 [6].

For parent-related factors, the questionnaire assessed both parents’ average daily ST. Parental self-efficacy in reducing their child’s ST was evaluated using a single question rated on a 5-point scale, ranging from “not at all confident” to “very confident.” Then the scores were divided into 3 categories based on percentiles [8,37]. Parents’ attitudes toward ST were evaluated by an 8-item questionnaire on a 5-point Likert scale, ranging from 1 (“strongly disagree”) to 5 (“strongly agree”), where scores above 32 indicated a positive attitude [8,10,37]. Additionally, parental perceptions were assessed using 11 questions that examined the effects of ST on their child’s physical, cognitive, and social well-being [43]. They rated the influence as 1 (“negative impact”), 2 (“no impact”), or 3 (“positive impact”), with higher scores indicating a more positive effect on their child’s specific well-being [37]. Parenting style was assessed using an adapted version of the Steinberg instrument, a widely used tool for measuring parenting style [44]. Two dimensions were measured: involvement (9 items) and strictness (6 items) on a 5-point scale from 1 (“strongly disagree”) to 5 (“strongly agree”). Based on the Baumrind classification, parents were categorized into 4 groups according to the median split of involvement and strictness [45]. Furthermore, parental restrictive practices on ST were assessed by 6 sedentary-related restrictive practice questions on a 5-point Likert scale, in which higher scores reflected more restrictive practices [46].

Home environment–related factors include information on the number of screen devices, the presence of screen devices in the child’s eating, playing, or sleeping rooms, and the presence of outdoor playthings. The neighborhood environment–related factors include questions about the availability of public facilities or a garden, perceived safety from crime, and perceived pedestrian safety.

Lastly, the outcome variable of this study, parental barriers to reducing a child’s excessive ST, was assessed using 6 items adapted from validated instruments [8,37]. Each item was rated on a 5-point Likert scale ranging from 1 (“strongly disagree”) to 5 (“strongly agree”). Item scores were averaged to produce a continuous barrier score ranging from 1 to 6, with higher values indicating stronger perceived barriers [8,37]. Internal consistency was confirmed with Cronbach α of 0.82, demonstrating good reliability.

### Data Quality Control

The data quality was meticulously ensured from the collection to the analysis stages. The standardized questionnaire was translated into Afan Oromo and Amharic, and then back into English. The researchers and the supervisor conducted regular checks for inconsistencies or missing data, and the data were coded and cleaned before analysis. Moreover, comprehensive training was provided to data collectors and supervisors on data collection tools, procedures, ethics, study aims, and participants’ rights.

### Data Processing and Analysis

EpiData (version 3.1; EpiData Association) was used for data entry and SPSS (version 20.0; IBM Corp) was used for analysis. All normality assumptions were tested and met for the continuous variables. Descriptive statistics were presented as frequency tables and in-text narratives. Dummy tables were created for all categorical variables, and bivariate linear regression analysis was conducted. All independent variables with *P*<.25 in the bivariate analysis were entered into the multiple linear regression model. Key assumptions were assessed and met: linearity was checked using scatterplots, homoscedasticity by examining residual plots, normality of residuals with histograms and the Shapiro-Wilk test, and multicollinearity using variance inflation factors (all <2). Statistical significance was set at *P*<.05, and unstandardized β coefficients with 95% CIs were reported.

### Ethical Considerations

The study was conducted in accordance with the Declaration of Helsinki. Accordingly, an ethical approval letter (reference number: IRB/23117/2023) was first obtained from the Arba Minch University Institutional Research Ethics Review Board Office. Then, a letter of support was submitted to the Woliso Town Administration to facilitate cooperation during data collection. After explicitly explaining the study’s aim, benefits, and risks, written informed consent was obtained from each participant. To maintain the confidentiality of participants’ information, personal identifiers, including names and house numbers, were anonymized.

## Results

### Sociodemographic Characteristics of Participants

A total of 423 parents took part in this study, resulting in a 100% response rate. The majority of participants were married (n=412, 97.4%), and three-quarters had a moderate (4-6) number of total family members (n=321, 75.9%). The mean ages of mothers and fathers were 31.29 (SD 5.511) and 37.32 (SD 5.939) years, respectively. Approximately half of the mothers (n=223, 52.7%) and the majority of fathers (n=343, 81.1%) completed college or higher education. In terms of employment, 188 (44.4%) mothers were unemployed, while most fathers, 415 (98.1%), were employed. Most participants were identified as Oromo, totaling 374 (88.4%) and 236 (55.8%) of participants reported following the Protestant religion. Around one-third (n=144, 34.0%) of participants earned less than 11,000 Ethiopian Birr (ETB 1=US $0.02 as of February 29, 2024), and one-quarter of participants lived in homes with 2 or fewer rooms (Table 1).

**Table 1.** Distribution of sociodemographic characteristics of under-five children parents in Woliso City Administration, Oromia, Ethiopia, 2024 (N=423).

Variables and category	Value, n (%)
Total family members	
≤3	24 (5.7)
4‐6	321 (75.9)
≥7	78 (18.4)
Mother’s age (y)	
<30	159 (37.6)
≥30	264 (62.4)
Father’s age (y)	
<30	44 (10.4)
≥30	379 (89.6)
Marital status	
Married	412 (97.4)
Separated	11 (2.6)
Mother’s education status	
No formal education	3 (0.7)
Primary education	42 (9.9)
Secondary education	155 (36.6)
College and above	223 (52.7)
Father’s education status	
No formal education	3 (0.7)
Primary education	32 (7.6)
Secondary education	45 (10.6)
College and above	343 (81.8)
Mother’s occupation	
Private sector	5 (1.2)
Public sector	155 (36.6)
Self-employed	75 (17.8)
Unemployed/housewife	188 (44.4)
Father’s occupation	
Private sector	19 (4.5)
Public sector	266 (62.9)
Self-employed	130 (30.7)
Unemployed	8 (1.9)
Religion	
Protestant	236 (55.8)
Orthodox	150 (35.5)
Muslim	37 (8.7)
Ethnicity	
Oromo	374 (88.4)
Amhara	19 (4.5)
Gurage	30 (7.1)
Number of rooms	
≤2	106 (25.1)
3‐5	254 (60.0)
≥6	63 (14.9)
Monthly income (ETB^a,b^)	
≤11,000	144 (34.0)
11,001‐20,000	182 (43.1)
≥20,001	97 (22.9)

a ETB: Ethiopian Birr.

b ETB 1=US $0.02 as of February 29, 2024.

### Child-Related Factors

In this study, the mean age of under-five children was 29.1 (SD 14.818) months. More than half of the children were male (n=233, 55.1%), and approximately one-fifth were an only child (n=97, 22.9%). Nearly half, 223 (52.7%), of the children received home care from their parents (Table 2). Additionally, the mean ST of under-five children in this study was 2.268 (SD 1.425) hours per day.

**Table 2.** Distribution of child-related characteristics among under-five children in Woliso City Administration, Oromia, Ethiopia, 2024 (N=423).

Variables and category	Value, n (%)
Age (mo)	
<24	156 (36.9)
≥24	267 (63.1)
Sex	
Male	233 (55.1)
Female	190 (44.9)
Number of children	
1	97 (22.9)
≥2	326 (77.1)
Childcare facility	
Home care by parents	223 (52.7)
Home care by other than parents	94 (22.2)
Childcare center	106 (25.1)

### Parental-Related Factors

The mean ST of mothers and fathers was 2.39 (SD 1.27) and 3.16 (SD 1.72) hours per day, respectively. In this study, approximately 190 (44.9%) of mothers had low self-efficacy to say no to their child’s ST. In addition, about one-third (n=129, 30.5%) of participants held a positive attitude toward their child’s ST. Regarding parenting style, approximately 165 (39.0%) participants were identified as neglectful (Table 3). The mean scores for parental perceptions of the effects of ST on children’s physical, cognitive, and social well-being were 6.95 (SD 2.31), 8.09 (SD 2.65), and 5.73 (SD 1.92), respectively. Moreover, the mean score of parental restrictive practice concerning ST was 19.74 (SD 6.45).

**Table 3.** Distribution of parental-related factors among parents of under-five children in Woliso City Administration, Oromia, Ethiopia, 2024 (N=423).

Variables and category	Value, n (%)
Mother’s ST^a^ (h)	
≤2	220 (52.0)
>2	203 (48.0)
Father’s ST (h)	
≤2	147 (34.8)
>2	276 (65.2)
Parental SE^b^	
Low	190 (44.9)
Moderate	56 (13.2)
High	177 (41.8)
Parental attitude	
Positive	129 (30.5)
Negative	294 (69.5)
Parenting style	
Authoritative	150 (35.5)
Authoritarian	83 (19.6)
Indulgent	25 (5.9)
Neglectful	165 (39.0)

a ST: screen time.

b SE: self-efficacy.

### Home Environment–Related and Neighborhood Environment–Related Factors

A significant majority of participants (n=401, 94.8%) had at least 3 screen devices in their homes, and approximately 197 (46.6%) parents placed a TV in the room where their child plays, eats, or sleeps. Additionally, 345 (81.6%) parents provided outdoor play equipment for their children, while only 122 (28.8%) parents reported the availability of public facilities, gardens, or playgrounds in their neighborhood (Table 4). Moreover, the mean scores for perceived safety concerning crime and pedestrian issues were 17.50 (SD 6.76) and 17.37 (SD 4.94), respectively.

**Table 4.** Distribution of home environment–related and neighborhood environment–related factors among mothers of under-five children in Woliso City Administration, Oromia, Ethiopia, 2024 (N=423).

Variables and category	Value, n (%)
Number of screen gadgets	
<3	22 (5.2)
≥3	401 (94.8)
Presence of screen device in child’s playing, eating, or sleeping room	
Yes	197 (46.6)
No	226 (53.4)
Do you provide outdoor plaything?	
Yes	345 (81.6)
No	76 (18.4)
Availability of public facilities	
Yes	122 (28.8)
No	301 (71.2)

### Barrier to Reducing Children’s ST

The overall mean score of barriers toward reducing their child’s ST was 4.28 (SD 1.04), with the most common challenges including the need for time to complete household chores, poor weather conditions, and the fact that their child enjoys screen-based activities (Table 5).

**Table 5.** Distribution of barriers to reducing children’s screen time among mothers of under-five children in Woliso City Administration, Oromia, Ethiopia, 2024 (N=423).

Barrier statement	Strongly disagree, n (%)	Disagree, n (%)	Somewhat agree, n (%)	Agree, n (%)	Strongly agree, n (%)	Mean (SD)
There is pressure from society to purchase and use media-related equipment.	112 (26.5)	27 (6.4)	128 (30.3)	40 (9.5)	116 (27.4)	3.05 (1.52)
My neighborhood is not safe for my child to play outdoors.	101 (23.9)	32 (7.6)	97 (22.9)	41 (9.7)	152 (35.9)	3.26 (1.58)
Poor weather limits my child’s opportunities to go outside.	35 (8.3)	25 (5.9)	129 (30.5)	54 (12.8)	180 (42.6)	3.75 (1.28)
I need a coping tool to meet demands of a busy day at work or raising multiple children.	76 (18.0)	27 (6.4)	73 (17.3)	46 (10.9)	201 (47.5)	3.64 (1.54)
I need time to do household chores.	62 (14.7)	20 (4.7)	45 (10.6)	47 (11.1)	249 (58.9)	3.95 (1.48)
My child really enjoys screen time activities.	47 (11.1)	31 (7.3)	93 (22.0)	55 (13.0)	197 (46.6)	3.77 (1.38)

### Bivariate Analysis of Barriers to Reducing a Child’s ST

The bivariate linear relationships between barrier to reducing ST and all independent variables, including sociodemographic, child-related, parental, home environment, and neighborhood environment factors, were illustrated in Table 6.

**Table 6.** Bivariate linear regression analysis of factors associated with barriers to reducing children’s screen time among mothers of under-five children in Woliso City Administration, Oromia, Ethiopia, 2024 (N=423).

Variables	β (95% CI)	SE	*P* value
Number of family members	−0.019 (−0.099 to 0.061)	0.041	.64
Mother’s age (y)	−0.015 (−0.033 to 0.003)	0.009	.11
Father’s age (y)	−0.017 (−0.033 to 0)	0.009	.05
Marital status (reference: married)			
Separated	1.184 (0.565 to 1.802)	0.315	<.001
Religion (reference: Protestant)			
Orthodox	−0.167 (−0.381 to 0.046)	0.109	.12
Muslim	0.264 (−0.098 to 0.626)	0.184	.15
Ethnicity (reference: Oromo)			
Amhara	0.179 (−0.305 to 0.663)	0.246	.47
Gurage	0.170 (−0.220 to 0.561)	0.199	.39
Mother’s educational status (reference: secondary school)			
No formal education	−0.188 (−1.379 to 1.004)	0.606	.76
Primary school	−0.416 (−0.772 to −0.060)	0.181	.02
College and above	−0.243 (−0.457 to 0.030)	0.109	.03
Mother’s occupation (reference: private sector)			
Public sector	0.165 (−0.764 to 1.095)	0.473	.73
Self-employed	0.339 (−0.606 to 1.284)	0.481	.48
Unemployed	0.456 (−0.471 to 1.383)	0.472	.33
Father’s educational status (reference: secondary school)			
No formal education	−0.089 (−1.317 to 1.139)	0.625	.89
Primary school	−0.220 (−0.696 to 0.256)	0.242	.36
College and above	−0.014 (−0.312 to 0.341)	0.166	.93
Father’s occupation (reference: private sector)			
Public sector	0.280 (−0.208 to 0.768)	0.248	.26
Self-employed	0.151 (−0.354 to 0.655)	0.257	.56
Unemployed	−0.138 (−1.004 to 0.728)	0.441	.75
Number of rooms in the house	0.006 (−0.057 to 0.068)	0.032	.86
Monthly income (ETB^a^^b,c^; reference: ≤11,000)			
11,001-20,000	−0.091 (−0.320 to 0.138)	0.117	.44
≥20,001	−0.199 (−0.469 to 0.071)	0.137	.15
Child’s age (mo)	0.004 (−0.003 to 0.010)	0.003	.28
Child’s sex (reference: male)			
Female	−0.124 (−0.325 to 0.077)	0.102	.22
Number of children	−0.041 (−0.119 to 0.036)	0.039	.29
Childcare setting (reference: home care by parents)			
Home care by others	−0.199 (−0.451 to 0.054)	0.129	.12
Childcare center	−0.021 (−0.264 to 0.221)	0.123	.86
Child’s ST^c^	0.650 (0.618 to 0.683)	0.017	<.001
Mother’s ST	0.097 (0.019 to 0.174)	0.040	.02
Father’s ST	−0.050 (−0.107 to 0.008)	0.029	.09
Self-efficacy to say no for ST (reference: high)			
Moderate	0.361 (0.050 to 0.671)	0.158	.02
Low	0.381 (0.169 to 0.593)	0.108	<.001
Parental attitude (reference: negative)			
Positive	0.451 (0.232 to 0.670)	0.111	<.001
Perception on the influence of ST on child’s physical well-being	0.041 (−0.020 to 0.084)	0.022	.06
Perception on the influence of ST on child’s cognitive well-being	0.046 (0.009 to 0.084)	0.019	.02
Perception on the influence of ST on child’s social well-being	0.029 (−0.023 to 0.081)	0.027	.27
Parenting style (reference: authoritative)			
Authoritarian	−0.035 (−0.315 to 0.246)	0.143	.81
Indulgent	0.344 (−0.099 to 0.787)	0.226	.13
Neglectful	0.158 (−0.073 to 0.390)	0.118	.18
Parental restrictive practices on ST	0.087 (0.074 to 0.101)	0.007	<.001
Number of screen devices at home (reference: <3)			
≥3	0.174 (−0.276 to 0.624)	0.229	.45
Screen device in child’s bedroom (reference: yes)			
No	−0.325 (−0.523 to −0.127)	0.101	.001
Outdoor play equipment (reference: yes)			
No	1.523 (1.256 to 1.791)	0.136	<.001
Availability of public facilities (reference: yes)			
No	1.739 (1.594 to 1.884)	0.074	<.001
Perceived safety related to crime	0.035 (0.021 to 0.049)	0.007	<.001
Perceived safety related to pedestrian	0.032 (0.012 to 0.052)	0.010	.002

a ETB: Ethiopian Birr.

b ETB 1=US $0.02 as of February 29, 2024.

c ST: screen time.

In the bivariate analysis, educational status of mothers and the availability of screen devices in the child’s bedroom were identified as having a negative association with parental barriers to reducing ST. Accordingly, mothers of under-five children who had primary education (β=−0.416, 95% CI −0.772 to −0.060; *P*=.02), mothers of under-five children with college education or above (β=−0.243, 95% CI −0.457 to −0.030; *P*=.03), and not having screen devices in the bedroom (β=−0.325, 95% CI −0.523 to −0.127; *P*=.001) were associated with fewer barriers.

On the other hand, factors found to be positively associated with parental barriers to reducing ST included marital separation (β=1.184, 95% CI 0.565 to 1.802; *P*<.001), child’s ST (β=0.650, 95% CI 0.618-0.683; *P*=.02), mother’s ST (β=0.097, 95% CI 0.019-0.174; *P*=.02), moderate maternal self-efficacy to say no to ST (β=0.361, 95% CI 0.050-0.671; *P*=.02), low maternal self-efficacy to say no to ST (β=0.381, 95% CI 0.169-0.593; *P*<.001), positive maternal attitude (β=0.451, 95% CI 0.232-0.670; *P*<.001), perception of the influence of ST on the child’s cognitive well-being (β=0.046, 95% CI 0.009-0.084; *P*=.02), restrictive practices regarding ST (β=0.087, 95% CI 0.074-0.101; *P*<.001), providing outdoor playthings (β=1.523, 95% CI 1.256-1.791; *P*<.001), presence of public facilities such as gardens and playgrounds (β=1.739, 95% CI 1.594-1.884; *P*<.001), perceived crime safety (β=0.035, 95% CI 0.021-0.049; *P*<.001), and perceived pedestrian safety (β=0.032, 95% CI 0.012-0.052; *P*=.002).

### Factors Associated With Barriers to Reducing a Child’s ST

In the simple linear regression analysis, 22 variables with a *P*<.25 were identified and included in the multiple linear regression models. All assumptions for multiple linear regression were met, and the explanatory variables in the final models explained about 89.4% of the variation in barriers to reducing ST (*R*²=0.894 and adjusted *R*²=0.892). The multiple linear regression analysis revealed that educational status of the mother, the child’s ST, attitude, perceptions of the effect of ST on cognitive well-being, restrictive practices regarding ST, and the availability of public facilities were significantly associated with barriers to reducing their under-five children’s ST.

In this study, mothers of under-five children with only a primary school education faced fewer barriers compared to those with a secondary school education (adjusted β=−0.130, 95% CI −0.255 to −0.004; *P*=.04). Additionally, participants with a positive attitude toward child ST were more likely to encounter higher barriers in reducing ST than those with a negative attitude (adjusted β=0.382, 95% CI 0.264-0.499; *P*<.001). For each unit increase in perceptions of the effect of ST on a child’s cognitive well-being, the mean score of barriers was increased by 0.021 (adjusted β=0.021, 95% CI 0.009-0.034; *P*=.001). Moreover, for a 1-unit increase in restrictive practices regarding ST, the mean score of barriers decreased by 0.011 (adjusted β=−0.011, 95% CI −0.004 to −0.018; *P*=.001). The only child-related factor that was significantly associated with parental barriers was the child’s ST. Accordingly, parents whose children spent more hours on screens faced greater barriers to reducing ST (adjusted β=0.369, 95% CI 0.328-0.409; *P*<.001). Lastly, parents who indicated a lack of public facilities, gardens, or playgrounds in their area were more likely to experience higher barriers to reducing ST than their counterparts (adjusted β=0.849, 95% CI 0.759-0.940; *P*<.001) (Table 7).

**Table 7.** Factors associated with barriers to reducing children’s screen time among mothers of under-five children in Woliso City Administration, Oromia, Ethiopia, 2024 (N=423).

Variables	Adjusted β (95% CI)	SE	*P* value
Mother’s educational status (reference: secondary school)			
Primary school	−0.130 (−0.255 to −0.004)	0.064	.04
Parental attitude toward ST^a^ (reference: negative)			
Positive	0.382 (0.264 to 0.499)	0.060	<.001
Parental perceptions of influence of ST on child’s cognitive well-being	0.021 (0.009 to 0.034)	0.006	.001
Parental restrictive practice on ST	−0.011 (−0.004 to −0.018)	0.003	.001
Child’s ST	0.369 (0.328 to 0.409)	0.021	<.001
Availability of public facilities, garden, and playthings (reference: yes)			
No	0.849 (0.759 to 0.940)	0.046	<.001

a ST: screen time.

## Discussion

### Principal Findings

This study assessed barriers to reducing ST and its associated factors among mothers of under-five children in the Woliso City Administration. Accordingly, the study found that the mean barrier score was 4.28 (SD 1.04). Factors significantly associated with barriers to reducing ST included mother’s educational status, child’s ST, attitudes, perceptions of the effect of ST on cognitive well-being, restrictive practices regarding ST, and the availability of public facilities.

In this study, the mean score for barriers was found to be 4.28 (SD 1.04), which is notably higher than the mean score 3.51 (SD 0.83) reported in a previous Malaysian study [37]. The variation in scores could be attributed to differences in study populations, as this study focused solely on mothers, while the Malaysian study included both parents [37]. Research indicates that mothers often bear a larger share of managing children’s routines and ST [47], and a recent systematic review also confirms that maternal caregiving roles remain central in moderating child ST [48]. Additionally, the difference in study settings may account for this variation. The nature of community-based studies, which reach a diverse population, often allows them to capture a broader range of everyday challenges and barriers experienced by parents. Socioeconomic status [37], cultural norms, and parenting practices [49] also likely contribute to these differences.

According to this study, mothers of under-five children with only a primary school education faced fewer parental barriers to reducing their children’s ST compared with those with a secondary school education. This finding may be because mothers with higher education levels may have greater access to resources, support systems, and outdoor employment status, which can influence their ability to manage their children’s ST effectively. This finding is congruent with research indicating that higher educational attainment often correlates with increased awareness of the potential negative impacts of ST, leading to more restrictive practices and fewer perceived barriers [50].

Additionally, mothers with a positive attitude toward ST were more likely to encounter higher barriers in reducing ST. This finding is supported by a study from Australia [49] and Malaysia [37]. Research indicates that parents’ positive attitudes toward ST can lead to more permissive practices and a lower perceived need to restrict ST, thereby increasing the barriers they face in attempting to reduce it [49]. Conversely, parents with negative attitudes are more likely to implement stricter controls and face fewer barriers. Recent reviews also highlight similar findings, noting that parental attitudes and self-efficacy strongly influence barriers to ST reduction [49].

Furthermore, our study reported that there was a positive association between parental perceptions of the effects of ST on their child’s cognitive well-being and barriers. This finding is supported by research indicating that parents who perceive ST as having a positive impact on cognitive development may face greater barriers [49]. When parents perceived ST to be beneficial to their child or themselves, it became a barrier for them to place necessary restrictions on ST activity [50,51]. This aligns with updated evidence showing that parental confusion and mixed perceptions about ST benefits remain a major barrier [48].

Apart from that, parental restrictive practices regarding ST were negatively associated with barriers to reducing ST. Parents who monitor and employ greater restrictions on ST may face fewer challenges in managing their child’s ST due to the consistent enforcement of ST rules [49,52]. This finding is congruent with recent intervention studies that report that restrictive practices are effective strategies for reducing barriers [53].

Furthermore, our study reported that mothers whose children spent more time on screens faced greater barriers to reducing ST. This was supported by previous studies [8,37,54]. Increased ST can lead to more ingrained habits and screen addiction [3], making it extremely hard for parents to enforce restrictions, which can increase parental barriers [55].

Finally, our study reported that parents who indicated a lack of public facilities, gardens, or playgrounds in their area faced greater barriers to reducing ST. The absence of accessible outdoor spaces limits children’s opportunity for physical activity, making it more challenging for parents to encourage alternatives to ST, thereby increasing barriers to reducing ST [56]. The UNESCO MGIEP (United Nations Educational, Scientific, and Cultural Organization—Mahatma Gandhi Institute of Education for Peace and Sustainable Development) highlighted that equitable access to green spaces is crucial for promoting healthy behaviors and reducing ST among children [57]. Additionally, community programs aimed at reducing ST were less successful in neighborhoods with limited green spaces, as children had fewer options for outdoor play [56].

These findings should be understood within Ethiopia’s social environment, where childcare facilities such as kindergartens and nursery schools are limited, and playgrounds and safe outdoor spaces are scarce. Such contextual realities restrict opportunities for alternative activities and create barriers to reducing screen use. The broader social environment, including limited facilities and cultural caregiving practices, remains crucial for interpreting and designing locally tailored interventions.

### Strengths and Limitations of the Study

To the best of our knowledge, this is a pioneering study in Ethiopia that identified barriers to reducing ST. Thus, it can be used as a baseline for future studies. Its community-based design ensured that diverse real-life challenges were captured. Comprehensive data collection through a validated questionnaire and a multilingual approach (Afan Oromo and Amharic) facilitated accurate responses. However, several limitations should be noted. First, the cross-sectional design limits causal inference. Second, reliance on self-reported ST may introduce recall bias, as parents may not accurately remember or estimate their child’s daily screen use. Third, social desirability bias may have influenced responses, with some parents underreporting ST or overstating restrictive practices to align with perceived norms. The reliance on maternal reports of their partner’s ST is also a limitation. Fourth, although we adjusted for multiple covariates, residual confounding cannot be ruled out, as unmeasured factors (eg, parental mental health) may also influence barriers. These limitations should be considered when interpreting the findings, and future longitudinal studies using objective measures of ST are recommended.

### Implications for Practice

The findings from this study underscore the need for targeted interventions to reduce ST among young children. Health care providers, educators, and policymakers should prioritize enhancing access to public facilities, gardens, and playgrounds as alternatives to ST. Additionally, implementing educational programs for parents can raise awareness of the negative impacts of excessive ST and provide strategies for effective management. Community-based initiatives that create screen-free zones and organize family-friendly events can foster environments that support healthy screen habits. Health care professionals should integrate ST management guidelines into routine pediatric care, offering parents consistent support and personalized recommendations. Providing parents with practical tools and resources for monitoring and restricting ST can help to overcome identified barriers and promote healthier screen habits among children.

For the scientific community, this study highlights the importance of further research on the barriers and predictors of parental efforts to reduce ST in various cultural and socioeconomic settings. It encourages the development and testing of community-based initiatives and interventions that create supportive environments for reducing children’s ST. Additionally, it advocates for policies that enhance access to public facilities, gardens, and playgrounds to promote healthy child development. By addressing a significant research gap, this pioneering study in Ethiopia provides valuable insights and potential solutions for both clinical practice and the scientific community, ultimately contributing to the global effort to manage and reduce children’s ST.

### Conclusions

Most mothers with a child aged younger than 5 years experienced 4 barriers in their efforts to reduce ST. In addition, this study identified several associated factors with barriers to reducing ST, including maternal education, parental attitudes, restrictive practices, perceptions of ST’s cognitive effects, the child’s ST, and lack of public facilities such as gardens and playgrounds. Based on these findings, we recommend that relevant bodies enhance access to public facilities and playgrounds to provide alternatives to screen use, implement educational programs for parents to raise awareness about the negative impacts of excessive ST, and develop community-based initiatives that support screen-free activities. Interventions should also address parental attitudes and misconceptions, promote balanced and evidence-based perspectives, and support consistent restrictive practices such as monitoring and removing screens from children’s bedrooms. Furthermore, community-based initiatives that encourage screen-free activities can complement household efforts, and integrating ST management guidelines into routine pediatric care will ensure that parents receive culturally appropriate support. Together, these measures can reduce barriers and foster healthier screen habits among young children.

## Abbreviations

ETB: Ethiopian Birr
ST: screen time
UNESCO MGIEP: United Nations Educational, Scientific, and Cultural Organization—Mahatma Gandhi Institute of Education for Peace and Sustainable Development
WHO: World Health Organization
